# The genome sequence of the Common Blue, *Polyommatus icarus *(Rottemburg, 1775)

**DOI:** 10.12688/wellcomeopenres.18772.1

**Published:** 2023-02-13

**Authors:** Konrad Lohse, Wellcome Sanger Institute Tree of Life programme, Wellcome Sanger Institute Scientific Operations: DNA Pipelines collective, Tree of Life Core Informatics collective, Roger Vila, Darwin Tree of Life Consortium

**Affiliations:** 1 Institute of Ecology and Evolution, University of Edinburgh, Edinburg, UK; 2 Institut de Biologia Evolutiva (CSIC - Universitat Pompeu Fabra), Barcelona, Spain

**Keywords:** Polyommatus icarus, Common Blue, genome sequence, chromosomal, Lepidoptera

## Abstract

We present a genome assembly from an individual male *Polyommatus icarus* (the Common Blue; Arthropoda; Insecta; Lepidoptera; Lycaenidae). The genome sequence is 512 megabases in span. Most of the assembly is scaffolded into 23 chromosomal pseudomolecules, including the assembled Z chromosome. The mitochondrial genome has also been assembled and is 15.6 kilobases long. Gene annotation of this assembly on Ensembl identified 13,350 protein-coding genes.

## Species taxonomy

Eukaryota; Metazoa; Ecdysozoa; Arthropoda; Hexapoda; Insecta; Pterygota; Neoptera; Endopterygota; Lepidoptera; Glossata; Ditrysia; Papilionoidea; Lycaenidae; Polyommatinae; *Polyommatus*; *Polyommatus icarus* (Rottemburg, 1775) (NCBI:txid265386).

## Background

The Common Blue, *Polyommatus icarus* (Rottemburg, 1775), is one of the most widespread Lycaenid butterflies in Europe, with a distribution ranging from the Mediterranean to northern Scandinavia, and from the Atlantic coast eastwards across temperate Asia. The species has also been introduced in several locations in Canada (it was first detected in 2005 in Montreal) and has expanded its range rapidly since ( D’Ercole *et al.*, 2022). 

The Common Blue is listed as a species of Least Concern both on the IUCN Red List of Europe ( van Swaay *et al.*, 2010) and the revised Red List for the UK ( Fox *et al.*, 2022). While *P. icarus* has declined in distribution in the UK in the 20th century ( León-Cortés *et al.*, 1999), it has increased in abundance over the last decade ( Fox *et al.*, 2022). Northern populations of the Common Blue, as well as those at the highest altitudes, are univoltine, those in central Europe and the south of the UK have two generations, and populations in the south of its range have three or even four generations per year. The species lives in a variety of grassland habitats. While the main foodplant of *P. icarus* in the UK is Bird's-foot trefoil ( *Lotus corniculatus*), the species is a generalist and can use a wide range of Fabaceae species, in the genera *Lotus*, *Medicago*, *Trifolium*, *Melilotus*, *Ononis*, *Genista*, *Astragalus*, *Onobrychis*, etc. Larvae are facultatively tended by ants of the genera *Lasius*, *Formica*, *Myrmica* and *Plagiolepis*.

The Common Blue exhibits considerable phenotypic variation and is known to hybridise with several other species, including *P. eros* ( Descimon & Mallet, 2009). Females generally have brown wings, but some have basal metallic blue scales, in some cases covering most of the wings as in the males. Remarkably, this female form is most frequent in some populations in the north-west of the UK and Ireland. Phylogeographic analyses of *P. icarus* have documented several highly diverged mitochondrial clades in Europe, some of which correspond to distinct *Wolbachia* strains ( Arif *et al.*, 2021; Dapporto *et al.*, 2022; Dincă *et al.*, 2011). A recent study of genomic variation revealed surprising levels of population structure within the UK ( Arif *et al.*, 2021), which may suggest a complex colonisation history for the UK.

The Common Blue has a haploid chromosome number of 23 ( Lorkoviç, 1990), and its genome size has been estimated as 482 Mb using flow cytometry ( Mackintosh *et al.*, 2019). Here we present a chromosomally complete genome sequence for *P. icarus*, based on a male specimen from Yellowcraig, Scotland.

### Genome sequence report

**Figure 1.**
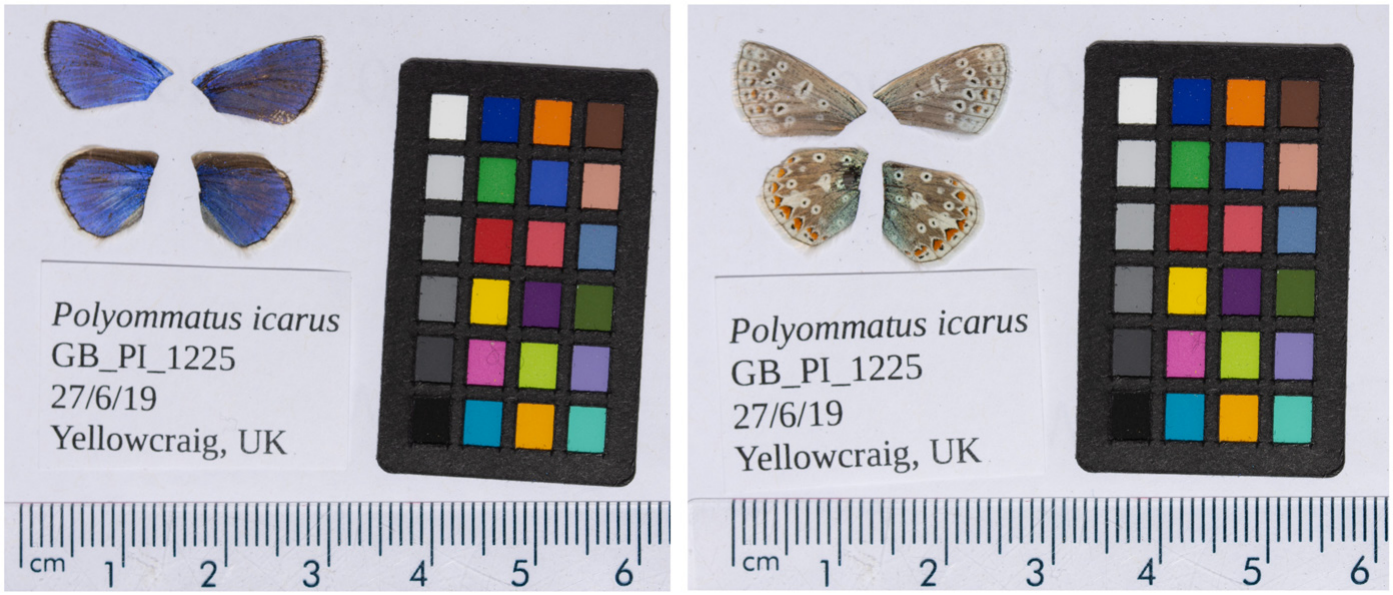
Forewings and hindwings of the male *Polyommatus icarus* (ilPolIcar1) specimen from which the genome was sequenced. Dorsal (left) and ventral (right) surface view of wings from specimen GB_PI_1225 from Yellowcraig, Scotland, used to generate Pacific Biosciences and 10X genomics data.

The genome was sequenced from one male *P. icarus* specimen ( Figure 1) collected in Yellowcraig, Scotland (latitude 56.06, longitude –2.77). A total of 35-fold coverage in Pacific Biosciences single-molecule HiFi long reads and 64-fold coverage in 10X Genomics read clouds was generated. Primary assembly contigs were scaffolded with chromosome conformation Hi-C data. Manual assembly curation corrected 30 missing joins or mis-joins and removed four haplotypic duplications, reducing the assembly length by 1.03% and the scaffold number by 29.79%, and increasing the scaffold N50 by 1.82%.

**Figure 2.**
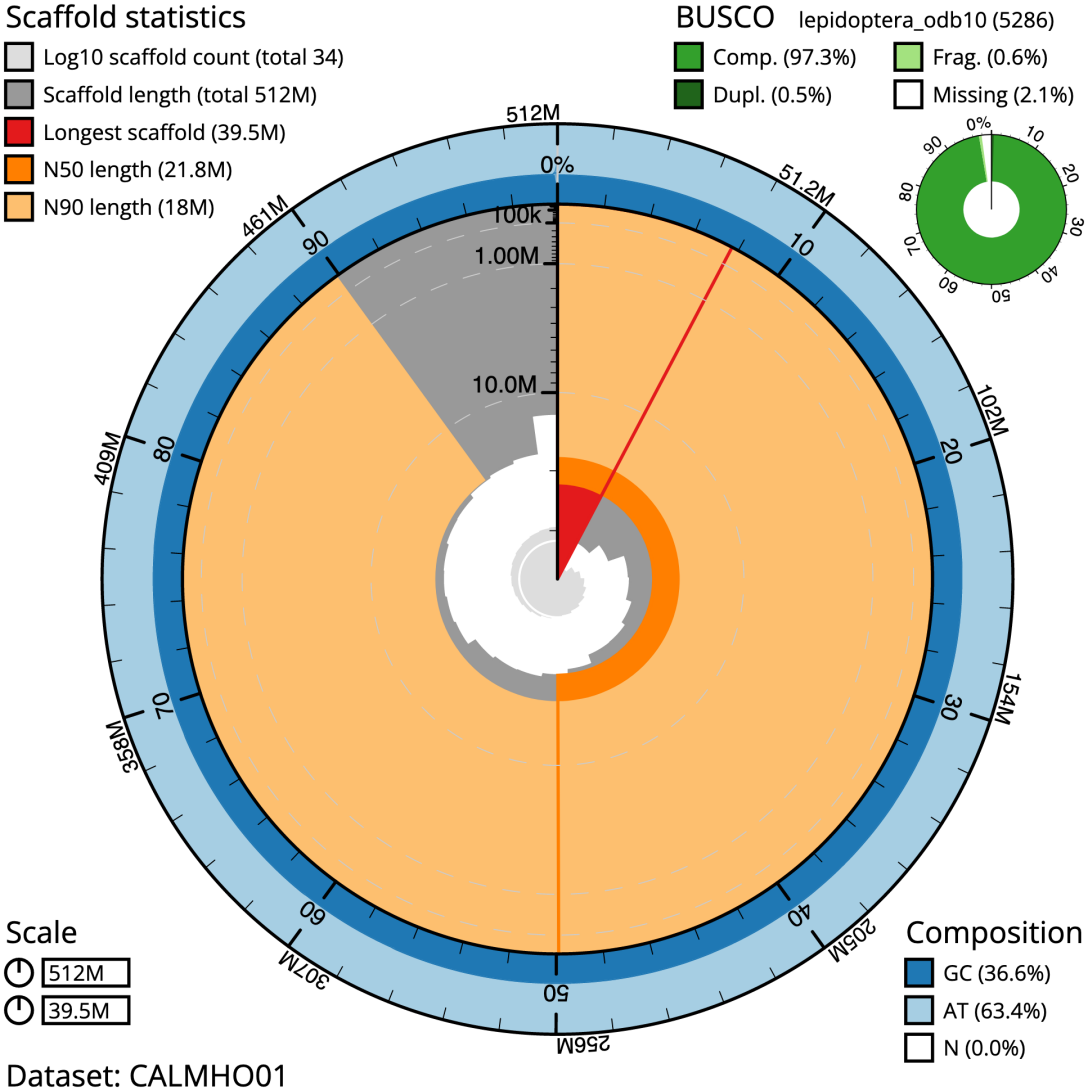
Genome assembly of *Polyommatus icarus*, ilPolIcar1.1: metrics. The BlobToolKit Snailplot shows N50 metrics and BUSCO gene completeness. The main plot is divided into 1,000 size-ordered bins around the circumference with each bin representing 0.1% of the 511,758,028 bp assembly. The distribution of scaffold lengths is shown in dark grey with the plot radius scaled to the longest scaffold present in the assembly (39,537,790 bp, shown in red). Orange and pale-orange arcs show the N50 and N90 scaffold lengths (21,845,677 and 17,972,970 bp), respectively. The pale grey spiral shows the cumulative scaffold count on a log scale with white scale lines showing successive orders of magnitude. The blue and pale-blue area around the outside of the plot shows the distribution of GC, AT and N percentages in the same bins as the inner plot. A summary of complete, fragmented, duplicated and missing BUSCO genes in the lepidoptera_odb10 set is shown in the top right. An interactive version of this figure is available at https://blobtoolkit.genomehubs.org/view/ilPolIcar1.1/dataset/CALMHO01/snail.

The final assembly has a total length of 511.7 Mb in 33 sequence scaffolds with a scaffold N50 of 21.8 Mb ( Table 1). Most (99.9%) of the assembly sequence was assigned to 23 chromosomal-level scaffolds, representing 22 autosomes, and the Z sex chromosome. Chromosome-scale scaffolds confirmed by the Hi-C data are named in order of size. ( Figure 2– Figure 5; Table 2). The assembly has a BUSCO v5.3.2 ( Manni *et al.*, 2021) completeness of 97.3% (single 96.8%, duplicated 0.5%) using the lepidoptera_odb10 reference set ( *n* = 5,286). While not fully phased, the assembly deposited is of one haplotype. Contigs corresponding to the second haplotype have also been deposited.

**Table 1.** Genome data for *Polyommatus icarus*, ilPolIcar1.1.

Project accession data	
Assembly identifier	ilPolIcar1.1
Species	*Polyommatus icarus*
Specimen	ilPolIcar1
NCBI taxonomy ID	265386
BioProject	PRJEB51269
BioSample ID	SAMEA7523143
Isolate information	ilPolIcar2 (Hi-C)
Raw data accessions	
PacificBiosciences SEQUEL II	ERR9081703
10X Genomics Illumina	ERR9123828, ERR9123829, ERR9123830, ERR9123831
Hi-C Illumina	ERR9123832, ERR9123833
Genome assembly	
Assembly accession	GCA_937595015.1
*Accession of alternate haplotype*	GCA_937629555.1
Span (Mb)	511.7
Number of contigs	137
Contig N50 length (Mb)	5.5
Number of scaffolds	33
Scaffold N50 length (Mb)	21.8
Longest scaffold (Mb)	39.54
BUSCO * genome score	C:97.3%[S:96.8%,D:0.5%], F:0.6%,M:2.1%,n:5,286
Genome annotation	
Number of protein-coding genes	13,350
Non-coding genes	2,894
Gene transcripts	26,748

* BUSCO scores based on the lepidoptera_odb10 BUSCO set using v5.3.2. C = complete [S = single copy, D = duplicated], F = fragmented, M = missing, n = number of orthologues in comparison. A full set of BUSCO scores is available at https://blobtoolkit.genomehubs.org/view/ilPolIcar1.1/dataset/CALMHO01/busco.

**Figure 3.**
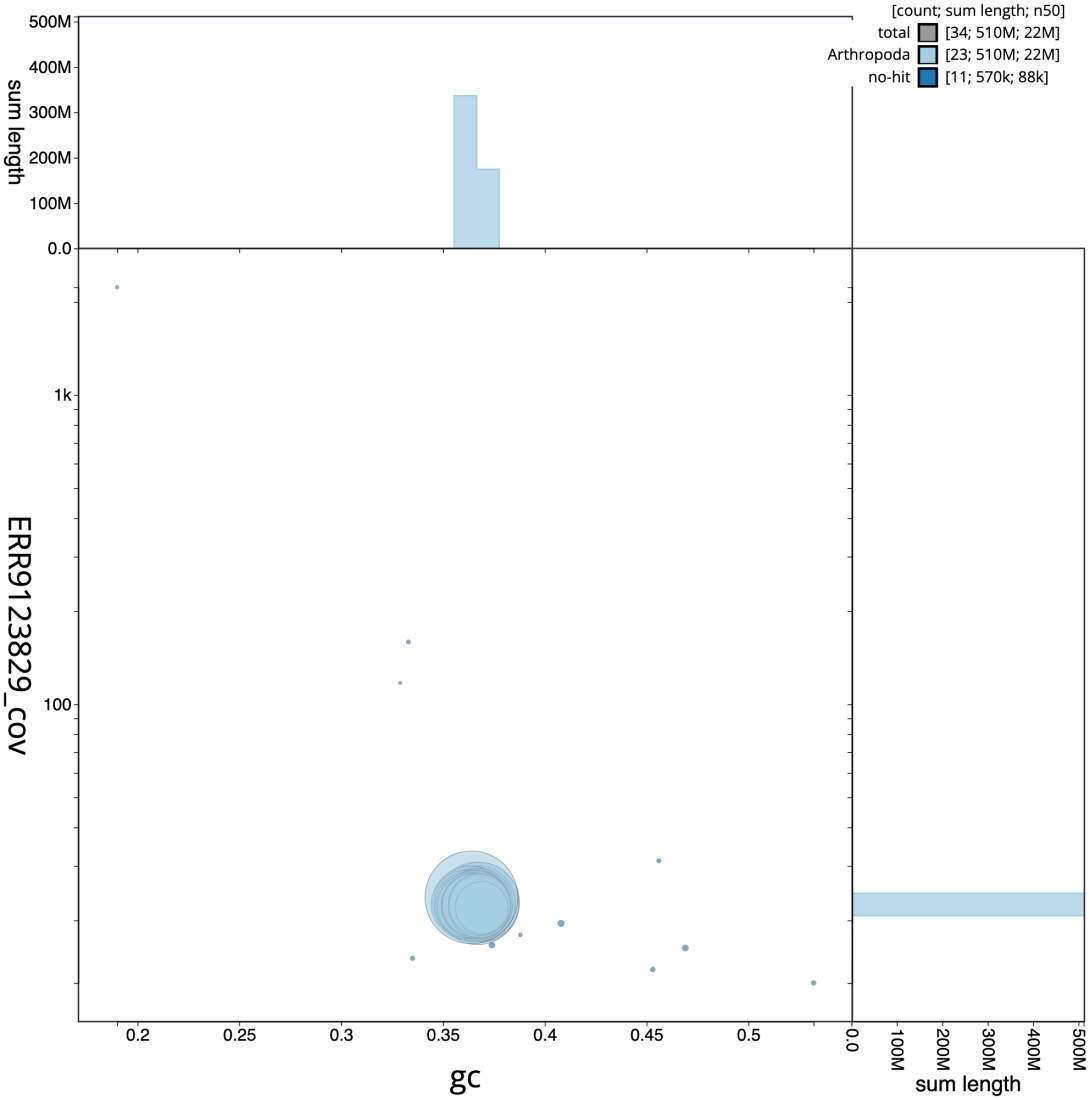
Genome assembly of *Polyommatus icarus*, ilPolIcar1.1: GC coverage. BlobToolKit GC-coverage plot. Scaffolds are coloured by phylum. Circles are sized in proportion to scaffold length. Histograms show the distribution of scaffold length sum along each axis. An interactive version of this figure is available at https://blobtoolkit.genomehubs.org/view/ilPolIcar1.1/dataset/CALMHO01/blob.

**Figure 4.**
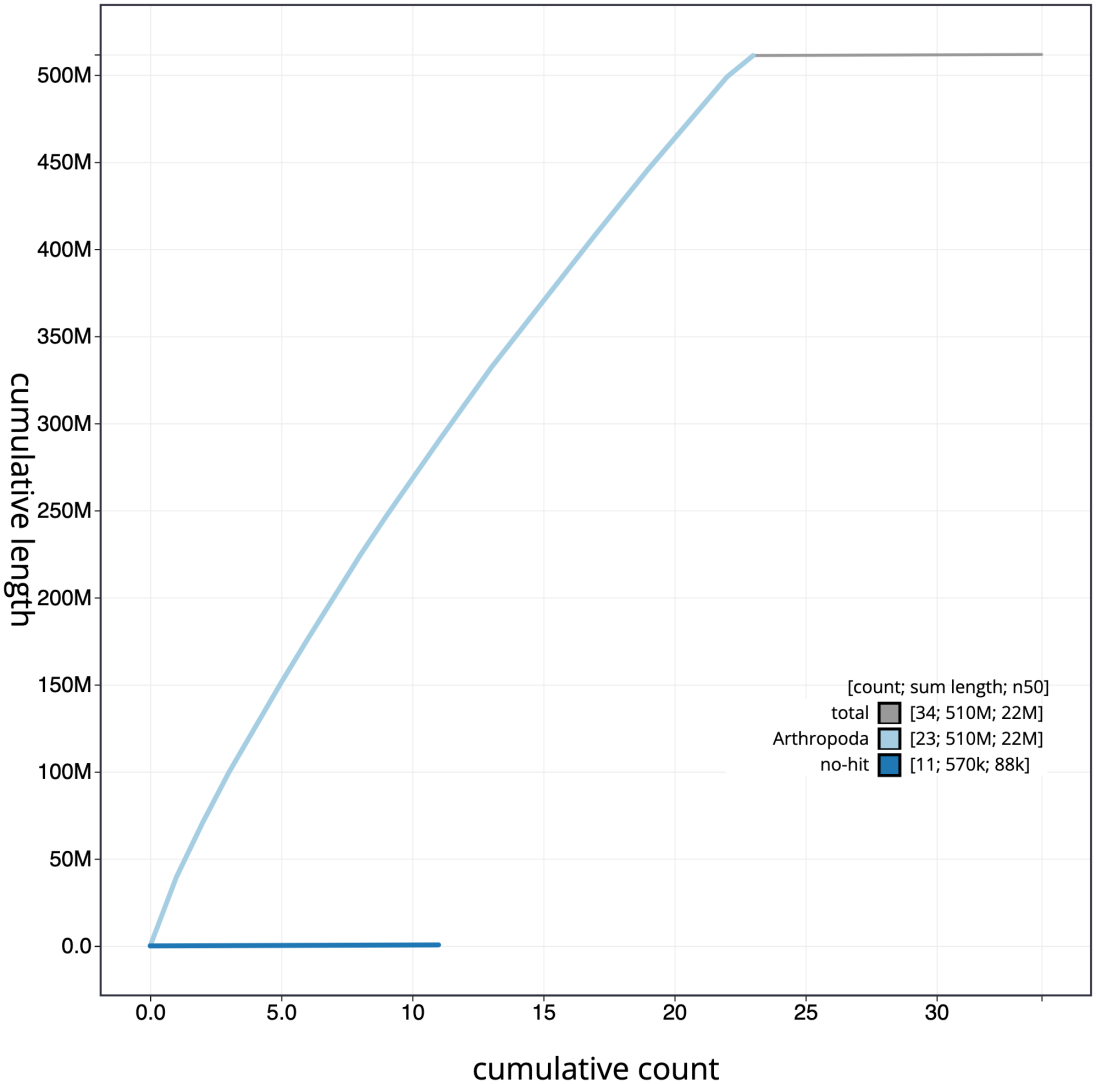
Genome assembly of *Polyommatus icarus*, ilPolIcar1.1: cumulative sequence. BlobToolKit cumulative sequence plot. The grey line shows cumulative length for all scaffolds. Coloured lines show cumulative lengths of scaffolds assigned to each phylum using the buscogenes taxrule. An interactive version of this figure is available at https://blobtoolkit.genomehubs.org/view/ilPolIcar1.1/dataset/CALMHO01/cumulative.

**Figure 5.**
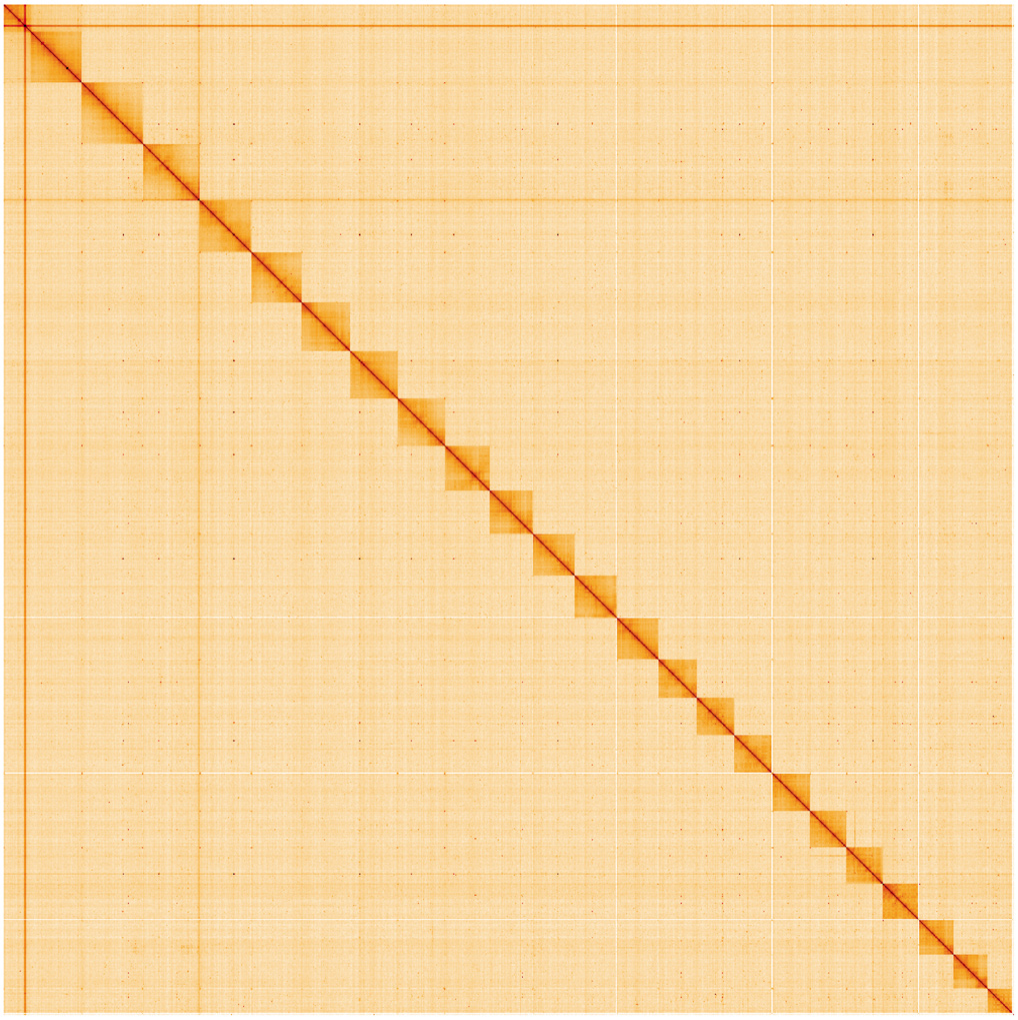
Genome assembly of *Polyommatus icarus*, ilPolIcar1.1: Hi-C contact map. Hi-C contact map of the ilPolIcar1.1 assembly, visualised using HiGlass. Chromosomes are shown in order of size from left to right and top to bottom. An interactive version of this figure may be viewed at https://genome-note-higlass.tol.sanger.ac.uk/l/?d=eNUJSOOCRMugElFi5deGdQ.

**Table 2.** Chromosomal pseudomolecules in the genome assembly of *Polyommatus icarus*, ilPolIcar1.

INSDC accession	Chromosome	Size (Mb)	GC%
OW569321.1	1	31.19	36.7
OW569322.1	2	28.84	36.8
OW569323.1	3	25.92	36.7
OW569324.1	4	25.74	36.4
OW569325.1	5	24.74	36.3
OW569326.1	6	24.06	36.5
OW569327.1	7	24.01	36.6
OW569328.1	8	22.58	36.7
OW569329.1	9	21.85	36.6
OW569330.1	10	21.32	36.6
OW569331.1	11	21.22	36.1
OW569332.1	12	20.83	36.6
OW569333.1	13	19.52	36.6
OW569334.1	14	19.29	36.1
OW569335.1	15	19.06	36.3
OW569336.1	16	18.98	36.6
OW569337.1	17	18.66	36.7
OW569338.1	18	18.58	36.5
OW569339.1	19	17.97	36.5
OW569340.1	20	17.68	36.8
OW569341.1	21	17.25	36.8
OW569342.1	22	12.36	36.9
OW569320.1	Z	39.54	36.4
OW569343.1	MT	0.02	19.1
-	unplaced	0.56	41.9

### Genome annotation report

The *P. icarus* genome assembly (GCA_937595015.1) was annotated using the Ensembl rapid annotation pipeline ( Table 1; https://rapid.ensembl.org/Polyommatus_icarus_GCA_937595015.1/). The resulting annotation includes 26,748 transcribed mRNAs from 13,350 protein-coding and 2,894 non-coding genes.

## Methods

### Sample acquisition and nucleic acid extraction

Two male *P. icarus* specimens (ilPolIcar1 and ilPolIcar2) were collected in Yellowcraig, Scotland, UK (latitude 56.06, longitude –2.77). The specimens were collected during the daytime with a net by Konrad Lohse (University of Edinburgh), who also identified the specimens. The specimens were preserved by snap-freezing at –80°C.

DNA was extracted at the Tree of Life laboratory, Wellcome Sanger Institute (WSI). The ilPolIcar1 sample was weighed and dissected on dry ice. Tissue from the whole organism was cryogenically disrupted to a fine powder using a Covaris cryoPREP Automated Dry Pulveriser, receiving multiple impacts. High molecular weight (HMW) DNA was extracted using the Qiagen MagAttract HMW DNA extraction kit. Low molecular weight DNA was removed from a 20 ng aliquot of extracted DNA using 0.8X AMpure XP purification kit prior to 10X Chromium sequencing; a minimum of 50 ng DNA was submitted for 10X sequencing. HMW DNA was sheared into an average fragment size of 12–20 kb in a Megaruptor 3 system with speed setting 30. Sheared DNA was purified by solid-phase reversible immobilisation using AMPure PB beads with a 1.8X ratio of beads to sample to remove the shorter fragments and concentrate the DNA sample. The concentration of the sheared and purified DNA was assessed using a Nanodrop spectrophotometer and Qubit Fluorometer and Qubit dsDNA High Sensitivity Assay kit. Fragment size distribution was evaluated by running the sample on the FemtoPulse system.

### Sequencing

Pacific Biosciences HiFi circular consensus and 10X Genomics read cloud DNA sequencing libraries were constructed according to the manufacturers’ instructions. DNA sequencing was performed by the Scientific Operations core at the WSI on Pacific Biosciences SEQUEL II (HiFi) and HiSeq X Ten (10X) instruments. Hi-C data were also generated from the ilPolIcar2 specimen using the Arima v2 kit and sequenced on the HiSeq X Ten instrument.

### Genome assembly

Assembly was carried out with Hifiasm ( Cheng *et al.*, 2021) and haplotypic duplication was identified and removed with purge_dups ( Guan *et al.*, 2020). One round of polishing was performed by aligning 10X Genomics read data to the assembly with Long Ranger ALIGN, calling variants with freebayes ( Garrison & Marth, 2012). The assembly was then scaffolded with Hi-C data ( Rao *et al.*, 2014) using YaHS ( Zhou *et al.*, 2022). The assembly was checked for contamination as described previously ( Howe *et al.*, 2021). Manual curation was performed using HiGlass ( Kerpedjiev *et al.*, 2018) and Pretext ( Harry, 2022). The mitochondrial genome was assembled using MitoHiFi ( Uliano-Silva *et al.*, 2021), which performed annotation using MitoFinder ( Allio *et al.*, 2020). The genome was analysed and BUSCO scores generated within the BlobToolKit environment ( Challis *et al.*, 2020). Table 3 contains a list of all software tool versions used, where appropriate.

**Table 3.** Software tools and versions used.

Software tool	Version	Source
BlobToolKit	3.2.9	Challis *et al.*, 2020
freebayes	1.3.1-17-gaa2ace8	Garrison & Marth, 2012
Hifiasm	0.16.1-r375	Cheng *et al.*, 2021
HiGlass	1.11.6	Kerpedjiev *et al.*, 2018
Long Ranger ALIGN	2.2.2	https://support.10xgenomics.com/genome-exome/ software/pipelines/latest/advanced/other-pipelines
MitoHiFi	2	Uliano-Silva *et al.*, 2021
PretextView	0.2	Harry, 2022
purge_dups	1.2.3	Guan *et al.*, 2020
YaHS	yahs-1.1.91eebc2	Zhou *et al.*, 2022

### Genome annotation

The Ensembl gene annotation system ( Aken *et al.*, 2016) was used to generate annotation for the *P. icarus* assembly (GCA_937595015.1). Annotation was created primarily through alignment of transcriptomic data to the genome, with gap filling via protein to-genome alignments of a select set of proteins from UniProt ( UniProt Consortium, 2019).

### Ethics/compliance issues

The materials that have contributed to this genome note have been supplied by a Darwin Tree of Life Partner. The submission of materials by a Darwin Tree of Life Partner is subject to the Darwin Tree of Life Project Sampling Code of Practice. By agreeing with and signing up to the Sampling Code of Practice, the Darwin Tree of Life Partner agrees they will meet the legal and ethical requirements and standards set out within this document in respect of all samples acquired for, and supplied to, the Darwin Tree of Life Project. Each transfer of samples is further undertaken according to a Research Collaboration Agreement or Material Transfer Agreement entered into by the Darwin Tree of Life Partner, Genome Research Limited (operating as the Wellcome Sanger Institute), and in some circumstances other Darwin Tree of Life collaborators.

## Data Availability

European Nucleotide Archive: *Polyommatus icarus* (common blue). Accession number PRJEB51269; https://identifiers.org/ena.embl/PRJEB51269 ( Wellcome Sanger Institute, 2022). The genome sequence is released openly for reuse. The *Polyommatus icarus* genome sequencing initiative is part of the Darwin Tree of Life (DToL) project. All raw sequence data and the assembly have been deposited in INSDC databases. Raw data and assembly accession identifiers are reported in Table 1.

## Author information

Members of the Darwin Tree of Life Barcoding collective are listed here: https://doi.org/10.5281/zenodo.4893703.

Members of the Wellcome Sanger Institute Tree of Life programme are listed here: https://doi.org/10.5281/zenodo.4783585.

Members of Wellcome Sanger Institute Scientific Operations: DNA Pipelines collective are listed here: https://doi.org/10.5281/zenodo.4790455.

Members of the Tree of Life Core Informatics collective are listed here: https://doi.org/10.5281/zenodo.5013541.

Members of the Darwin Tree of Life Consortium are listed here: https://doi.org/10.5281/zenodo.4783558.
