# Supplementary material for: The genome sequence of the Common Blue, Polyommatus icarus (Rottemburg, 1775)
Source: Wellcome Open Res. 2023 Feb 13;8:72. [Version 1] doi: 10.12688/wellcomeopenres.18772.1 (PMC11824579; doi:10.12688/wellcomeopenres.18772.1)
Supplement: Peer review history

# Reviewer report: Reviewer response for version 1

**DOI:** 10.21956/wellcomeopenres.20814.r70603

**Published:** 2024-06-07

**Authors:** Josephine Paris

**Affiliations:** 1 Department of Life and Environmental Sciences, Polytechnic University of Marche, Ancona, Marche, Italy

This data note presents a chromosome-level genome assembly of the Common Blue butterfly. The report is well-written and clearly described. The Background section sets the scene very nicely in regards to the species' biology, ecology, conservation context, and topics of interest which may make use of the new genome assembly. Protocols are good, and the Methods section is clear. The data accession is present and functional. I have no major points, and only minor considerations to improve clarity:

 - In the abstract, I suggest a more quantitative measure of how much (i.e. 99.9%) of the assembly was assigned to the 23 chromosomes, rather than “most”

 - Please specify how much Hi-C data was generated

 - For the annotation, please expand further on what sort of transcriptomic data was used

Are sufficient details of methods and materials provided to allow replication by others?

Yes

Is the rationale for creating the dataset(s) clearly described?

Yes

Are the datasets clearly presented in a useable and accessible format?

Yes

Are the protocols appropriate and is the work technically sound?

Yes

Reviewer Expertise:

ecological genomics, molecular ecology

I confirm that I have read this submission and believe that I have an appropriate level of expertise to confirm that it is of an acceptable scientific standard.

# Reviewer report: Reviewer response for version 1

**DOI:** 10.21956/wellcomeopenres.20814.r70608

**Published:** 2024-05-25

**Authors:** Francesco Cicconardi

**Affiliations:** 1 School of Biological Sciences, University of Bristol, Bristol, England, UK

The article is a summary of the genome assembly of *Polyommatus icarus.* The assembly appears to be of good quality using appropriate methods (HiFi long reads, 10X reads, Hi-C data to confirm scaffolding, manual curation of the final assembly) and following the high standard of the Darwin Tree of Life Project.

 This is not the first report I have reviewed. The quality of the data is very good. My only concern is about the transparency of the methods. In my personal opinion, they are too succinct. There's no indication of how they run the different bioinformatic tools. That is a missed opportunity to in the first place be transparent, and second to guide the scientific community on how to perform these analyses.

Are sufficient details of methods and materials provided to allow replication by others?

No

Is the rationale for creating the dataset(s) clearly described?

Yes

Are the datasets clearly presented in a useable and accessible format?

Yes

Are the protocols appropriate and is the work technically sound?

Yes

Reviewer Expertise:

Genomics

I confirm that I have read this submission and believe that I have an appropriate level of expertise to confirm that it is of an acceptable scientific standard, however I have significant reservations, as outlined above.

# Reviewer report: Reviewer response for version 1

**DOI:** 10.21956/wellcomeopenres.20814.r66961

**Published:** 2023-09-20

**Authors:** James Mallet

**Affiliations:** 1 Harvard University, Cambridge,, United States, USA

Summary: the genome sequence of *Polyommatus icarus* is reported.

 The genome is going to be useful. The paper will be welcome to future users of the genome. The polyommatine blues are a particularly difficult group with many closely related species worldwide (although not so many in UK!), hybridization, and some crazy chromosome evolution in some species. 

 I like it that the biology was discussed, which makes a simple sequencing data paper like this more interesting for the reader! 

 The technical aspects discussed with information about coverage, technologies used, genome size, scaffolding, BUSCO scores and other information was all useful. I've not checked out the sequence data myself, but the summary statistics and information about Hi-C mapping all look good.

Are sufficient details of methods and materials provided to allow replication by others?

Yes

Is the rationale for creating the dataset(s) clearly described?

Partly

Are the datasets clearly presented in a useable and accessible format?

Yes

Are the protocols appropriate and is the work technically sound?

Yes

Reviewer Expertise:

Evolutionary genomics of butterflies

I confirm that I have read this submission and believe that I have an appropriate level of expertise to confirm that it is of an acceptable scientific standard.
